# Increased Numbers of Culturable Inhibitory Bacterial Taxa May Mitigate the Effects of *Batrachochytrium dendrobatidis* in Australian Wet Tropics Frogs

**DOI:** 10.3389/fmicb.2018.01604

**Published:** 2018-07-18

**Authors:** Sara C. Bell, Stephen Garland, Ross A. Alford

**Affiliations:** ^1^College of Science and Engineering, James Cook University, Townsville, QLD, Australia; ^2^College of Public Health, Medical and Veterinary Sciences, James Cook University, Townsville, QLD, Australia

**Keywords:** amphibian, cutaneous bacteria, *Batrachochytrium dendrobatidis*, microbiota, cell-free supernatant, inhibitory bacteria, chytridiomycosis, disease mitigation

## Abstract

Symbiotic bacterial communities resident on amphibian skin can benefit their hosts. For example, antibiotic production by community members can control the pathogen *Batrachochytrium dendrobatidis* (*Bd*) and it is possible for these community members to be used as probiotics to reduce infection levels. In the early 1990s, the emergence of *Bd* caused declines and disappearances of frogs in the Australian Wet Tropics; the severity of its effects varied among species and sites. Some species have since recolonized despite enzootic *Bd* within their populations. This variation in history among species and sites provided an opportunity to investigate the role of anti-fungal cutaneous bacteria in protecting frogs against *Bd* infection. We collected cutaneous swab samples from three species of frogs at two upland and two lowland sites in the Wet Tropics, and used *in vitro* challenge assays to identify culturable *Bd*-inhibitory bacterial isolates for further analysis. We sequenced DNA from cultured inhibitory isolates to identify taxa, resulting in the classification of 16 *Bd*-inhibitory OTUs, and determined whether inhibitory taxa were associated with frog species, site, or intensity of infection. We present preliminary results showing that the upper limit of *Bd* infection intensity was negatively correlated with number of inhibitory OTUs present per frog indicating that increased numbers of *Bd*-inhibiting taxa may play a role in reducing the intensity of *Bd* infections, facilitating frog coexistence with enzootic *Bd*. One upland site had a significantly lower prevalence of *Bd* infection, a significantly higher proportion of frogs with one or more culturable *Bd*-inhibitory OTUs, a greater number of inhibitory bacterial genera present per frog, and statistically significant clustering of individual frogs with similar *Bd*-inhibitory signatures when compared to all other sites. This suggests that *Bd*-inhibitory taxa are likely to be particularly important to frogs at this site and may have played a role in their ability to recolonize following population declines. Our findings suggest that the use of multi-taxon *Bd*-inhibitory probiotics to support at-risk amphibian populations may be more effective than single-taxon alternatives.

## Introduction

Antibiotic-producing bacterial symbionts can protect their hosts from disease (Currie et al., 1999; Haas and Défago, 2005; Scott et al., 2008; Mao-Jones et al., 2010; Mattoso et al., 2012). For example, a high proportion of bacteria isolated from healthy coral can produce antibiotic compounds active against coral pathogens (Ritchie, 2006; Mao-Jones et al., 2010; Zhang et al., 2013). However, the best-studied mutualisms exist between insects and their bacterial symbionts. Both fungus-farming ants and pine beetles house antibiotic-producing bacteria in specialized cuticular compartments to control pathogens that threaten their food supply (Currie et al., 1999, 2006; Scott et al., 2008). In addition, solitary digger wasps use antibiotic-producing bacteria to protect their cocooned larvae against pathogens (Kroiss et al., 2010), and fungus-farming ants maintain a bacterial biofilm on their cuticles to control a fungal pathogen (Mattoso et al., 2012). Variation in the bacterial strain present can cause differential morbidity in fungus-farming ants (Poulsen et al., 2010), demonstrating that antibiotic production by symbionts is important for host protection.

Antibiotic production by symbiotic bacteria is also important in vertebrates. In two bird species, the European Hoopoe and the Green Woodhoopoe, nestlings harbor antibiotic-producing bacteria in their uropygial glands to control feather-degrading pathogens (Soler et al., 2008; Martín-Vivaldi et al., 2010). Symbiotic bacterial communities resident on amphibian skin can also produce, antibiotic compounds that contribute to the control of *Batrachochytrium dendrobatidis* (*Bd*; Becker et al., 2009, 2015; Harris et al., 2009a,b; Lam et al., 2010; Kueneman et al., 2016), a pathogenic fungus responsible for worldwide amphibian declines (Berger et al., 1998; Stuart et al., 2004; Skerratt et al., 2007; Wake and Vredenburg, 2008).

One aspect of community ecology theory predicts that complex communities, with higher numbers of species and hence more potential interactions, are generally more resistant to invasion than simple communities with fewer species (Robinson and Valentine, 1979). This has been demonstrated in soil bacterial communities (Matos et al., 2005; van Elsas et al., 2012; Hol et al., 2015; Hu et al., 2016), and also in the locust gut (Dillon et al., 2005) where higher numbers of inoculated bacterial isolates have been associated with greater resistance against pathogens.

Studies of amphibian skin microbiota have produced similar results. *In vitro* studies have demonstrated the potential importance of multi-species bacterial communities in resistance to *Bd* (Loudon et al., 2014; Piovia-Scott et al., 2017). Co-cultured synthetic multi-species communities were more effective against *Bd* than single species alone (Piovia-Scott et al., 2017). While there are several possible explanations for this effect, the presence of greater numbers of *Bd*-inhibitory metabolites following co-culture of bacterial isolates can have a synergistic effect (Loudon et al., 2014). While no field studies have reported a reduction of *Bd* load with increased *Bd*-inhibitory isolate richness, increased Proteobacteria phylotype richness on the skin of *Eleutherodactylus coqui* frogs was correlated with reduced *Bd* infection loads (Longo et al., 2015). A large portion of *Bd*-inhibitory isolates described to date fall within this phylum (Woodhams et al., 2007; Lam et al., 2010; Walke et al., 2011; Flechas et al., 2012; Bell et al., 2013; Daskin et al., 2014; Becker et al., 2015; Holden et al., 2015; Madison et al., 2017).

Patterns of frog decline and recovery in the Australian Wet Tropics following the arrival of the pathogen *Bd* in the late 1980s (Laurance et al., 1996; Berger et al., 1999; McDonald and Alford, 1999) differed among species and sites. Frog populations of all species at lowland sites (below 400 m) did not appear to experience declines, while several species, including *Litoria nannotis* (waterfall frog) and *Litoria rheocola* (common mist frog), suffered either declines or local extirpation at all upland rainforest sites (above 400 m; Richards et al., 1993; McDonald and Alford, 1999). Some populations of these species have subsequently reappeared at some but not all sites from which they disappeared (McDonald et al., 2005; Woodhams and Alford, 2005; Skerratt et al., 2010; McKnight et al., 2017). This suggests multiple, potentially independent appearances of resistance to *Bd*, or loss of virulence by the pathogen (McKnight et al., 2017). Another species, *Litoria serrata* (green-eyed tree frog) appeared to suffer temporary population declines at upland sites, followed by recovery within a few years (Richards et al., 1993; McDonald and Alford, 1999; Richards and Alford, 2005).

The mechanisms allowing population recovery or recolonization are likely to vary among species and sites (McKnight et al., 2017). There is substantial genetic separation among Wet Tropics frog populations (Schneider et al., 1998; Cunningham, 2001; Hoskin et al., 2005; Richards et al., 2010; Bell et al., 2012); variation among populations in both innate and acquired immune defenses due to selection pressures imposed by the pathogen may affect host resistance to *Bd* and ultimately survival (Rollins-Smith et al., 2002, 2011; Woodhams et al., 2010; Savage and Zamudio, 2011; Fites et al., 2012, 2014; Bataille et al., 2015; McKnight et al., 2017; Voyles et al., 2018). Pathogen virulence may also vary among regions (Berger et al., 2005; Farrer et al., 2011; Becker et al., 2017). Therefore, resistance to *Bd* infection is likely to have been acquired independently at these sites.

Symbiotic cutaneous bacteria are considered to be an important component of the amphibian innate immune defense system (Woodhams et al., 2007, 2014; Harris et al., 2009a). It is likely that the suite of cutaneous bacteria present on amphibian skin can evolve broad antimicrobial activity against invading pathogens much faster than adaptation of the host’s innate and acquired defenses (Rosenberg et al., 2007; King et al., 2016; Rosenberg and Zilber-Rosenberg, 2016). Therefore it is possible that at least some of the recolonized frogs in the Wet Tropics uplands have developed resistance to *Bd* through acquisition or evolution of more effective cutaneous bacterial symbionts.

The patterns of frog reappearances in the Wet Tropics uplands provide an opportunity to investigate whether the anti-fungal cutaneous bacterial microbiota have played a role in the ability of frogs to recolonize upland sites. We describe the culturable anti-*Bd* cutaneous bacterial microbiota of three species of rainforest frogs from four sites in northern Queensland, Australia, and assess how its composition may contribute to the health and population recovery of frogs at sites with different histories of pathogen-related population declines.

## Materials and Methods

### Field Sites and Species

We collected cutaneous swab samples from the stream-dwelling rainforest frogs *L. serrata, L. nannotis*, and *L. rheocola* at four rainforest field sites in the Wet Tropics bioregion where enzootic *Bd* infection exists, with one upland and one lowland site in each of two latitudinally separated national parks (**Table 1**). *L. nannotis* are the most, and *L. serrata* the least, strongly associated with water, and *L. serrata* are the least susceptible to population fluctuations as a result of *Bd* infection (McDonald and Alford, 1999; Woodhams and Alford, 2005). The upland sites were subject to extensive frog population declines in the early 1990s, while frog populations at the lowland sites persisted. Sampling was conducted in winter, when *Bd* infection is typically more prevalent (Berger et al., 2004; Woodhams and Alford, 2005; Kriger and Hero, 2007; Sapsford et al., 2013), to maximize chances of including *Bd*-infected frogs in the study. This study was conducted in compliance with the National Health and Medical Research Council’s Australian Code of Practice for the Care and Use of Animals for Scientific Purposes, 7th Edition, 2004, the Queensland Animal Care and Protection Act, 2001 and the Queensland Nature Conservation Act, 1992. Approval was granted from James Cook University Animal Ethics Committee (A1316 and A1420) and the Queensland government (scientific purposes permit number WITK05922209).

**Table 1:** Locations, species and *Batrachochytrium dendrobatidis* infection status of frogs sampled for each survey site, and overall *Bd* infection prevalence by site.

Site	Species			*Bd*
	LS	LN	LR	prevalence
Windin Creek North (Upland), Wooroonooran National Park 17°22′01.6^′′^ S 145°42′58.3^′′^ E	2 (4)	9 (7)	5 (8)	0.53
Frenchmans Creek (Lowland), Wooroonooran National Park 17°18′32.8^′′^ S 145°55′04.2^′′^ E	2 (4)	3 (9)	5 (9)	0.33
Kirrama Bridge 11 Creek (Upland), Murray Upper National Park 18°12′49.9^′′^ S 145°47′52.9^′′^ E	1 (10)	0 (10)	–	0.05
Kirrama Bridge 8 Creek (Lowland), Murray Upper National Park 18°11′44.8^′′^ S 145°52′05.4^′′^ E	1 (10)	4 (10)	0 (8)	0.17

*Numbers indicate the number of infected individuals present from the 10 frogs of each species sampled at each site and, in parentheses, the number of individuals from which the isolates used in this study were obtained. Species are Litoria serrata (LS), L. nannotis (LN), and L. rheocola (LR).*

### Collection of Bacterial Samples

We hand-captured frogs from vegetation and rocks bordering the creeks, in the winters of 2009 and 2010. All handling was carried out with a new plastic bag and pair of vinyl gloves for each animal to prevent contact with bacteria from our skin, and to preclude the transfer of pathogens or symbionts between animals. We gently restrained each animal by hand and rinsed it twice with a stream of sterile distilled water from a wash bottle to remove transient bacteria, which can differ from the resident microbiota (Lauer et al., 2007). We then swabbed over the dorsal and ventral skin from knee to neck five times using sterile rayon swabs (MW112, MW&E, Bath UK) moistened with sterile distilled water, and immediately transferred the swab’s contents to a low-nutrient agar plate (R2A, BD, Franklin Lakes, NJ, United States) by rotating the swab on the plate surface. Agar plates were sealed with parafilm (Parafilm “M,” Pechiney Plastic Packaging, Inc., Chicago, IL, United States), held at ambient temperature (10–25°C) and returned to the laboratory within 72 h. We collected an additional swab sample (MW100, MW&E, Bath UK) by rotating the swab over the abdomen, hands, feet, and thighs twice for analysis of *Bd* infection status.

### Isolation, Purification, and Storage of Microbial Cultures

Inverted R2A agar plates were incubated for 48–72 h at ambient temperature (22–25°C) in the laboratory until we observed microbial culture growth. We examined plates daily for 5 days using a dissecting microscope and selected all colonies with different morphological characteristics for isolation to pure (axenic) culture using standard microbiological techniques (Salle, 1961). When initial agar plates generated from swabs plated in the field did not yield any viable bacterial colonies, possibly due to media choice or the presence of potent antimicrobial peptides on the frogs, we excluded samples from those frogs from further analysis. Of a total of nine initial plates that produced no viable cultures, five were from *L. rheocola*, and four were from *L. nannotis*. In contrast, R2A agar plates originating from *L. serrata* at the upland and lowland sites in Wooroonooran National Park produced very large numbers of viable bacterial colonies making isolation difficult and time-consuming; all of these plates appeared visually very similar. Because time and available effort constrained the number of these plates we could fully sample, we selected agar plates haphazardly from only four *L. serrata* at each of the Wooroonooran sites (**Table 1**). For all other combinations of site and species, all plates with bacterial colonies present were included.

### Diagnosis of *Batrachochytrium dendrobatidis* Infection

DNA was extracted from skin swabs with PrepMan^TM^ Ultra (Applied Biosystems, Scoresby, VIC, Australia) and quantitative PCR (Real-time TaqMan^®^ assay) used to diagnose *Bd* infection status as per Boyle et al. (2004) with the addition of BSA (Garland et al., 2010). We considered frogs to be infected with *Bd* when at least two of the three technical PCR replicates were positive. *Bd* intensity was calculated as the mean of the technical replicates.

### Challenge Assays

We conducted challenge assays on cell free supernatants from axenic isolates, and categorized them as totally inhibitory, partially inhibitory, or non-inhibitory according to their effects on *Bd* growth as described by Bell et al. (2013). Briefly, cell-free supernatants for each isolate were obtained by centrifuging and filtering bacterial cultures incubated in TGhL medium (8 g tryptone, 1 g hydrolysed gelatin, 2 g lactose, to 1 L deionised water, autoclaved) at 23°C for 48 h. *Bd* isolate “Gibbo River, L. Les, 06-LB-1” was incubated on TGhL agar plates (as above recipe with 10 g bacteriological agar) at 23°C for 3 days. Zoospores were flushed from the plate with sterile TGhL medium, filtered to remove sporangia and resuspended in sterile TGhL at 2 × 10^6^ zoospores ml^-1^. We conducted challenge assays in 96-well microplates (Costar 3595, Corning) with five replicates of each sample (50 μl cell-free supernatant and 50 μl of *Bd* zoospores), positive, negative, and medium only controls. Plates were incubated at 23°C for approximately 7 days until maximum growth in the positive controls was observed. Daily absorbance readings were collected at 492 nm using a spectrophotometer (Multiskan Ascent, Thermo Scientific) and from these we calculated the proportion of inhibition observed in each sample on its maximum growth day relative to the positive control. Bacterial isolates that produced cell-free supernatants exhibiting total inhibition against *Bd* were selected for taxonomic identification by DNA sequencing. Due to financial constraints it was not possible to sequence other isolates.

### Bacterial DNA Extraction

We used a sterile toothpick to inoculate each axenic isolate into a 1.7 ml sterile microtube containing 400 μl molecular grade water. Samples were vortexed to create a cell suspension and subjected to three freeze-thaw cycles (70°C/-80°C; 10 min each), and then centrifuged at 7500 ×*g* for 5 min to pellet cell debris. We used the supernatant directly as template in the DNA amplification reaction, but if this was unsuccessful, DNA was extracted using a Qiagen DNeasy Blood and Tissue Kit (Qiagen, Doncaster, VIC, Australia) as per the manufacturer’s protocol.

### Amplification of 16S rRNA Gene

We amplified the 16S rRNA gene from pure bacterial isolates by PCR on Bio-Rad C1000/S1000 thermal cyclers (Bio-Rad, Hercules, CA, United States) with the bacteria-specific primer 8F (5′-AGAGTTTGATCCTGGCTCAG-3′) and the universal primer 1492R (5′-GGTTACCTTGTTACGACTT-3′) (Lane, 1991). The PCR reaction mix contained 0.2 μM of each primer, 0.2 mM dNTPs, 3 mM MgCl_2_, 0.2 mg ml^-1^ BSA, 1.25 U HotStar Taq polymerase (Qiagen, Doncaster, VIC, Australia) with 1× buffer and <1 ug template DNA. The thermocycling parameters were: 95°C for 15 min followed by 35 cycles of 94°C for 1 min, 48°C for 1 min, 72°C for 1.5 min, and a final extension for 10 min at 72°C.

### DNA Sequencing and Analysis

PCR product purification and Sanger DNA sequencing were conducted by Macrogen, Inc. (South Korea) using both forward and reverse primers described above. We aligned forward and reverse nucleotide sequences in Geneious Pro (Biomatters, Ltd.; Drummond et al., 2010) to create a consensus sequence of approximately 1400 bp. Following alignment of sequences, we compiled a FASTA file containing a single representative of each unique (one or more bp difference from any other sequence) consensus sequence and defined operational taxonomic units (OTUs) using mothur (v1.36.1; Schloss et al., 2009). Sequences were clustered at 96% sequence similarity to create genus-level OTUs and taxonomy was assigned using the SILVA reference database v128 (Quast et al., 2013; Yilmaz et al., 2014). When three or more sequences were present in a given OTU, the sequence used to assign taxonomy was the sequence that was the minimum distance to the other sequences in the OTU. Clustering was based on 96% sequence similarity to group the isolates into unique genera because our goal was to examine the diversity of cultured taxonomic groups likely to have different mechanisms of inhibiting the growth of *Bd*. Although some genera could comprise multiple OTUs that each produce different antifungal compounds, it is likely that some subgeneric OTUs would have the same mechanisms. For example, multiple *Pseudomonas* species can produce the antifungal compound 2,4-diacetylphloroglucinol (Gutierrez-Garcia et al., 2017). Splitting genera into multiple OTUs may overestimate the diversity of inhibitory mechanisms, so we chose the more conservative approach of leaving genera intact. We examined how well our sequenced data were likely to reflect the total number of culturable inhibitory bacterial OTUs present across all frog taxa at all of our sampling sites by constructing a species accumulation curve using the program EstimateS (Colwell, 2013). Because our study was primarily aimed at examining how the composition of culturable bacterial assemblages on individual frogs affected their *Bd* infection status, we also examined whether the isolates we cultured from each individual were likely to have captured all culturable totally inhibitory OTUs present on their skins. We did this by plotting number of totally inhibitory OTUs found on each frog against the total number of cultured isolates (inhibitory and non-inhibitory) obtained from each frog, and fitting a species accumulation curve to this relationship.

We added additional 16S sequences from closely related bacterial type strains (SILVA reference database v123; Quast et al., 2013; Yilmaz et al., 2014) to our FASTA file sequences and conducted a SINA alignment (Pruesse et al., 2012). From this alignment, we constructed a neighbor-joining phylogenetic tree in Geneious Pro (Biomatters, Ltd.; Drummond et al., 2010) and visualized this in Figtree v1.4.3 (Rambaut, 2016).

### Data Manipulation and Analysis

As data were not normally distributed, and attempts at normalization via transformation were unsuccessful, we used non-parametric statistical tests where applicable. All statistical analyses were carried out using R (v3.2.4)^1^ and figures produced using *ggplot2* (Wickham and Chang, 2016). We excluded *L. rheocola* from most of the analyses to balance species across sites, but included them in the graphics when appropriate. By including only the two species that were present at all sites, we minimized the possibility that site effects could reflect differences between species.

We used a generalized linear model (GLM) with a binomial response to compare prevalence of infection in *L. serrata* and *L. nannotis* (the species that occurred at all sites) across sites. We used Tukey’s *post hoc* pairwise comparisons (Tukey, 1949) to examine differences among pairs of sites using the function “glht” in the package *multcomp* (Hothorn et al., 2017), and adjusted *p*-values using the false discovery rate method (Benjamini and Hochberg, 1995). We assessed significance of the effects in this and other GLMs using the “ANOVA” function from the package *car* (Fox et al., 2017). To determine whether the ability of bacterial cell-free supernatants to inhibit *Bd* differed with infection status, species and site (*L. serrata* and *L. nannotis* only), we used permutational multivariate analysis of variance (PERMANOVA) on the proportions of isolates from each frog (*L. serrata* and *L. nannotis* only) that were in each of two challenge assay result categories; totally inhibitory and partially inhibitory. For this, we used the function “adonis2” with marginal terms (variables were assessed in the model together to show their impact when added last) in the community ecology package *vegan* (Oksanen et al., 2017). Tests were performed with 10000 permutations using Euclidean distance as the distance measure. We used the function “pairwise.adonis” (Martinez Arbizu, 2016) with Benjamini and Hochberg corrected *p*-values to examine differences among site pairs.

Following OTU classification, when totally inhibitory isolates were present on *Bd*-infected frogs, we used quantile regression at the 90th percentile level to examine the relationship between the intensity of *Bd* infection and the number of OTUs present on each frog using the package *quantreg* (Koenker et al., 2017) with 1000 bootstrap replications, and calculated pseudo-R^2^ (Koenker and Machado, 1999). We used quantile regression because it is recommended for use when examining relationships in which the independent variable sets a boundary for the dependent variable, but the value the dependent variable takes within that boundary is affected by many other factors (Cade and Noon, 2003). This is the case for *Bd* infection intensity, which can be affected by time since exposure, recent history of body temperature, and effects of innate and inducible immune responses not related to bacteria, among other things. We used the 90th percentile because we were interested in whether inhibitory bacteria set an upper boundary, and the 90th percentile was the highest we could practically examine given the number of observations in our data set. Frogs that did not carry any totally inhibitory isolates were excluded from our quantile regressions because the goal of these analyses was to examine how the upper limit of intensity of infection responds as number of inhibitory taxa increases. Frogs with no inhibitory OTUs will necessarily have no upper limit set by their microbiota, and are thus in a separate class, not comparable for our purpose to frogs with one or more inhibitory OTUs.

If inhibitory bacterial assemblages have evolved in response to *Bd*, they might differ across sites due to the differential availability of candidate sets of bacteria. Similarly, each frog species is likely to provide a different substrate for microbes to colonize. However, we would expect them to be similar across individuals within sites and species. Therefore, to determine if individual frogs clustered according to the taxonomy of their *Bd*-inhibitory bacterial microbiota within sites, frog species or with their interaction, we used PERMANOVA as described above with the Bray–Curtis distance measure (Bray and Curtis, 1957) with 10000 permutations. We excluded *L. rheocola* to ensure that species were balanced across sites. When the interaction term was not significant we repeated the analysis using only the two main effects. We checked for heterogeneity of multivariate dispersion using function “betadisper” in package *vegan* (Oksanen et al., 2017) with 10000 permutations. We used the function “pairwise.adonis” (Martinez Arbizu, 2016) with Benjamini and Hochberg corrected *p*-values to examine differences among pairs of sites. When clustering was observed, we visualized results with non-metric multidimensional scaling (nMDS) using the function “metaMDS.” When the nMDS failed to converge, we identified samples that were disconnected using the function “disconnected.” As a result, we removed three samples that each had just one unique *Bd*-inhibitory OTU from the nMDS analyses.

We used a GLM, specifying that the response came from a Poisson distribution, to examine species and site-specific differences in the number of *Bd*-inhibitory cutaneous bacterial OTUs present on frogs. We used Tukey’s *post hoc* pairwise comparisons (Tukey, 1949) to examine differences among site pairs as above with *p*-values adjusted using the false discovery rate method (Benjamini and Hochberg, 1995). For this, we excluded all data from *L. rheocola* to ensure that species were balanced across sites, and analyzed data as species-site pairs to test whether one species was responsible for any site-specific effects observed. Finally, we used a Fisher’s Exact Test to examine the association between the proportion of frogs with *Bd*-inhibitory OTUs and field site; we did not use a GLM to examine the effects of site and species simultaneously because numbers of individuals from which any OTUs were isolated was as low as 4 for some species-site combinations, so that the statistical power of a full analysis would have been near zero. Because this analysis excluded “species” as a factor, any species effects could have been confounded with site effects, since the numbers of each species from which OTUs were isolated differed among sites. To examine this possibility, we conducted a separate Fisher’s Exact Test to determine whether there were across-site effects of species (*L. nannotis* and *L. serrata* only, as these were the species in the site analysis) on the proportion of frogs with *Bd*-inhibitory OTUs.

## Results

### Infection Status of Frogs

Thirty-two of the 110 frogs (29%) sampled were infected with *Bd* (**Table 1**). Our GLM on data for the two species (*L. nannotis* and *L. serrata*) that were present at all sites indicated that the prevalence of infection differed significantly between those species (ANOVA: χ^2^ = 7.77, df = 1, *p* = 0.005) and among sites (ANOVA; χ^2^ = 14.98, df = 3, *p* = 0.0002); the interaction effect was not significant (ANOVA; χ^2^ = 7.50, df = 3, *p* = 0.058). *Post hoc* comparisons using Benjamini and Hochberg adjusted *p*-values showed that the Wooroonooran upland and Kirrama upland sites differed significantly (*p* = 0.019). Two of the five remaining pairwise comparisons were suggestive (Wooroonooran upland vs. Kirrama lowland and Wooroonooran upland vs. Wooroonooran lowland, *p* = 0.0892 for both cases). The remaining pairwise comparisons were not significantly different (*p* > 0.100 in all cases). Prevalence in *L. nannotis* and *L. serrata* combined (the species common to all sites) was 5% at Kirrama Uplands, 25% at Kirrama Lowlands, 25% at Wooroonooran Lowlands, and 55% at Wooroonooran Uplands.

We could not statistically compare intensity of infection among species within each site due to the low numbers of infected frogs. However, of the 29 infected frogs, 13 had infections of 10 zoospore equivalents or less indicating relatively low infection levels. Higher infection intensities occurred almost exclusively in *L. rheocola* at the Wooroonooran sites.

### Culture and Challenge Assays

**FIGURE 1:**
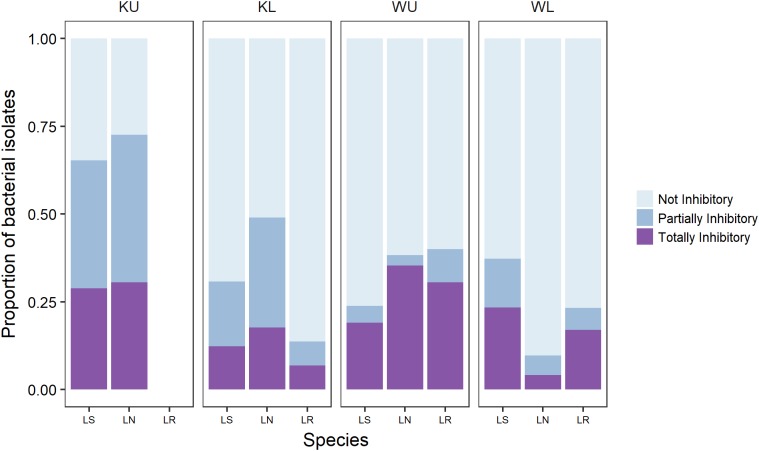
The proportion of culturable isolates as categorized by challenge assay results for each frog species at each site. Category definitions follow Bell et al. (2013). Species are *Litoria serrata* (LS), *L. nannotis* (LN), and *L. rheocola* (LR). Sites are Kirrama upland (KU), Kirrama lowland (KL), Wooroonooran upland (WU), and Wooroonooran lowland (WL).

We sampled bacterial isolates from a total of 89 frogs (**Table 1**). We obtained a total of 1005 bacterial isolates across all frog species and sites, with a mean of 11.3 cultured bacterial isolates per frog (Range = 1–36, *SD* = 8.3). This is broadly in line with the number of isolates reported in other studies (Woodhams et al., 2007; Walke et al., 2011, 2015a; Becker et al., 2015). Challenge assay results showed that 68.5% of frogs from which isolates were obtained had one or more isolates with some activity against *Bd*; 55% had totally inhibitory isolates and 58% had partially inhibitory isolates [following the definition of Bell et al. (2013); **Figure 1**].

Our PERMANOVA analysis of data for *L. nannotis* and *L. serrata*, the species that occurred at all sites, showed that the proportion of isolates in each category of inhibition (totally inhibitory and partially inhibitory) did not depend significantly upon infection status or species (PERMANOVA: infection status, pseudo-*F*_1,58_ = 0.478, *p* = 0.628, species, pseudo-*F*_1,58_ = 0.880, *p* = 0.422), but differed significantly among sites (**Figure 1**; PERMANOVA; pseudo-*F*_3,58_ = 6.189, *p* < 0.001). *Post hoc* comparisons among sites using Benjamini and Hochberg adjusted *p*-values showed that frogs at the Kirrama upland site had a significantly greater proportion of *Bd*-inhibitory isolates than those at all other sites (*p* < 0.01 in all cases). Frogs at the Wooroonooran upland and Kirrama lowland sites also differed significantly (*p* = 0.0285). The remaining site-pairs were not significantly different from each other (*p* > 0.100 for both cases).

### Identification of Bacterial Isolates

**FIGURE 2:**
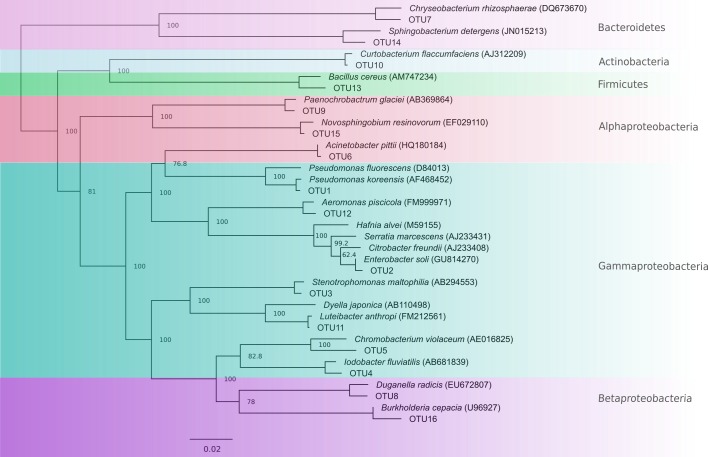
Neighbor-joining tree showing OTUs with representative type species for comparison. Nodes are labeled with percentage bootstrap support values. Labels with 100% bootstrap support were removed from some nodes to improve visualization.

From a total of 156 totally inhibitory isolates sequenced, we identified 105 unique 16S rRNA sequences. These comprised 16 OTUs at the 96% sequence similarity level, each representative of one genus (**Figure 2** and **Table 2**). 75% of OTUs fell within the Proteobacteria phylum with small contributions from the phyla Bacteriodetes, Actinobacteria, and Firmicutes, which is in line with the findings of other studies (Harris et al., 2006; Woodhams et al., 2007; Lam et al., 2010; Walke et al., 2011, 2015a; Flechas et al., 2012; Bell et al., 2013; Daskin et al., 2014; Becker et al., 2015; Holden et al., 2015; Madison et al., 2017). 16S rRNA gene sequences have been lodged in Genbank with accession numbers KJ191368–KJ191378, KJ191380–KJ191390, and MG491528–MG491661.

**Table 2:** Taxonomic classification of 16S rRNA OTUs by frog species and site.

			Number of totally inhibitory isolates within each OTU by species and site										
			KU		KL			WU			WL		
Taxonomy/representative genus		Sequences	LS	LN	LS	LR	LN	LS	LR	LN	LS	LR	LN
**Proteobacteria**									
Gammaproteobacteria									
OTU1	*Pseudomonas*	69	11	14	2	3	6	4	9	2	7	10	1
OTU2	*Enterobacter*	35	14	13	2	–	1	–	–	1	3	1	–
OTU3	*Stenotrophomonas*	21	1	6	2	–	1	3	3	–	3	2	–
OTU6	*Acinetobacter*	7	2	5	–	–	–	–	–	–	–	–	+
OTU12	*Aeromonas*	2	–	–	–	–	2	–	–	–	–	–	–
OTU11	*Luteibacter*	1	–	–	–	–	–	–	–	–	1	–	–
Betaproteobacteria								
OTU5	*Chromobacterium*	4	–	–	1	–	2	–	–	–	–	1	–
OTU4	*Iodobacter*	6	–	–	–	–	2	–	1	2	–	1	–
OTU8	*Duganella*	3	–	3	–	–	–	–	–	–	–	–	–
OTU16	*Burkholderia*	1	–	1	–	–	–	–	–	–	–	–	–
Alphaproteobacteria						
OTU9	*Paenochrobactrum*	1	–	–	–	–	1	–	–	–	–	–	–
OTU15	*Novosphingobium*	1	–	–	–	1	–	–	–	–	–	–	–
Bacteroidetes									
Flavobacteriia								
OTU7	*Chryseobacterium*	2	–	1	–	–	–	–	–	1	–	–	–
Sphingobacteriia								
OTU14	*Sphingobacterium*	1	–	1	–	–	–	–	–	–	–	–	–
Actinobacteria								
OTU10	*Curtobacterium*	1	–	–	–	–	–	–	–	–	1	–	–
Firmicutes							
OTU13	*Bacillus*	1	–	–	–	–	–	–	–	–	–	–	1
		Total	28	44	7	4	15	7	13	6	15	15	2

*Species are Litoria serrata (LS), L. nannotis (LN), and L. rheocola (LR). Sites are Kirrama upland (KU), Kirrama lowland (KL), Wooroonooran upland (WU), and Wooroonooran lowland (WL).*

### Analysis of OTU Diversity Across All Sites and Species and Upon Individual Frogs

**FIGURE 3:**
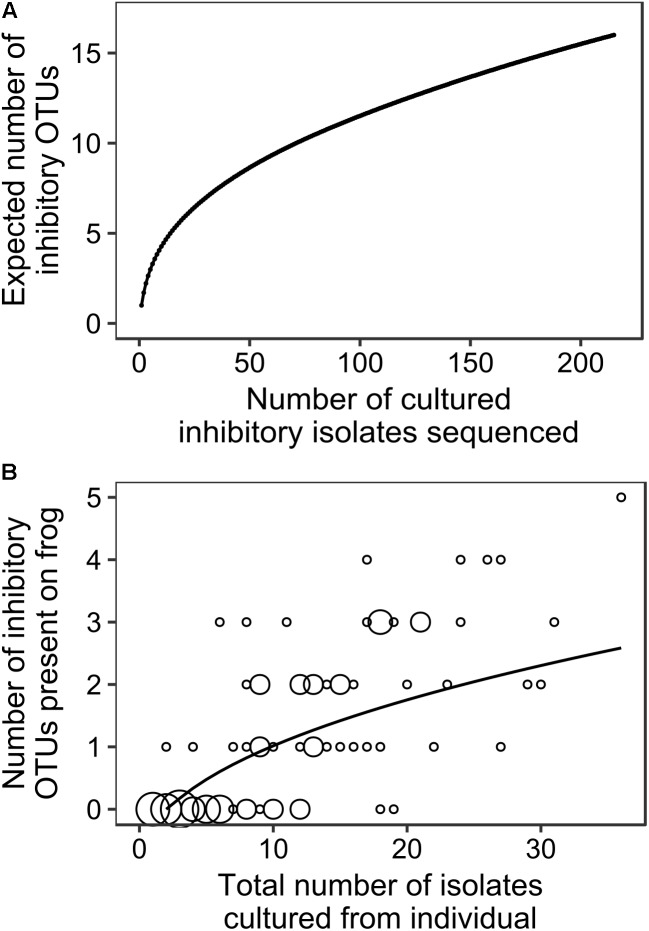
The relationships between **(A)** the number of totally inhibitory bacterial isolates identified and sequenced and the expected number of totally inhibitory OTUs (EstimateS; Colwell, 2013), and **(B)** the total number of isolates obtained from each individual frog and the number of totally inhibitory OTUs detected. The size of circles corresponds to the number of frogs at each combination of number of isolates and number of OTUs, with the smallest indicating one individual and the largest indicating eight. The best fit accumulation curve is a power curve fitted to the number of OTUs plus 1 (*y* = 0.717x^0.449^ with *R^2^* = 0.505, *p* = 0.001).

**Figure 3A** summarizes the relationship between total number of cultured inhibitory isolates sequenced and expected number of totally inhibitory OTUs obtained for our data aggregated across all species and sites. It shows that our sampling effort is not ideal because we typically sampled less than 30 isolates from any individual (**Figure 3B**). However, as we often sampled the majority of colonies that grew on the plates and in all cases we sampled all morphologically different isolates present, it is a limitation of culturing studies in general that it is impossible to attain adequate sampling effort. **Figure 3B** summarizes our analysis of the relationship between the total number of isolates we found on each individual frog and the number of unique cultured totally inhibitory OTUs included in those isolates. It provides additional information as it includes bacterial isolates that were sampled from agar plates that were not inhibitory. However, as our sampling effort is less than ideal due to the nature of culturing studies, our OTU-based analyses and results should be considered as preliminary findings.

### Number of Totally Inhibitory OTUs and *Bd* Infection Intensity

**FIGURE 4:**
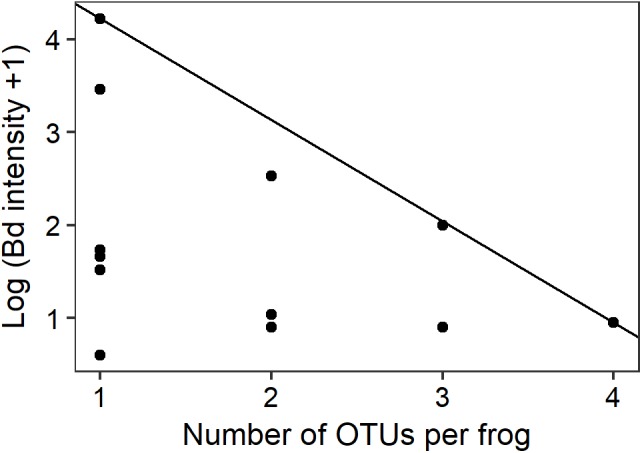
Intensity of *Bd* infection decreased as the number of inhibitory OTUs increased. The ninetieth percentile regression line shows the negative relationship between the number of OTUs per infected frog and the upper limits of *Bd* infection intensity (*n* = 14, *t* = -2.818, *p* = 0.0155).

Quantile regression at the 90th percentile level demonstrated a preliminary significant negative relationship between the number of *Bd*-inhibitory OTUs present per frog and the upper limits of *Bd* infection intensity (**Figure 4**; *n* = 14, *t* = -2.818, pseudo-R^2^ = 0.369, *p* = 0.0155). No frogs with highly intense *Bd* infections and high numbers of inhibitory bacteria were found during this study.

### Preliminary Species and Site-Specific Analyses

**FIGURE 5:**
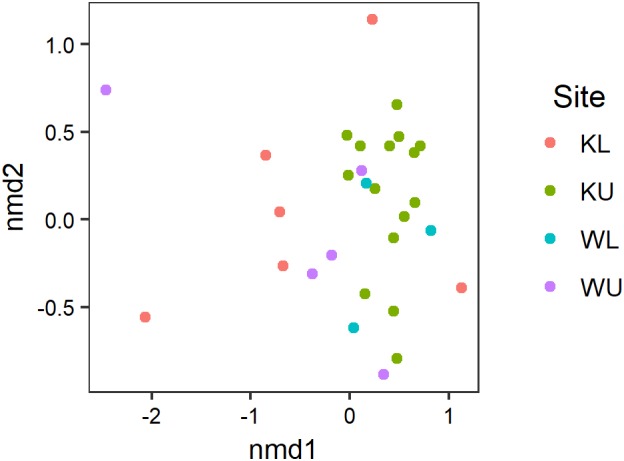
Results of an nMDS illustrating how the OTU signatures of frogs cluster by site. Stress = 0.124. Sites are KL, Kirrama lowland; KU, Kirrama upland; WL, Wooroonooran lowland; WU, Wooroonooran upland.

Our PERMANOVA examining the effects of species and site on the composition of *Bd*-inhibitory cutaneous bacterial OTUs showed no significant interaction between species and site (pseudo-*F*_3,31_ = 1.237, *p* = 0.221) and the effect of species was also not significant (pseudo-*F*_1,34_ = 0.661, *p* = 0.721), however, the effect of site was significant (pseudo-*F*_3,34_ = 1.780, *p* = 0.024). The multivariate dispersion of data using the Bray–Curtis distances used by the PERMANOVA was not significantly heterogeneous among sites (ANOVA; *F*_3,35_ = 2.541, *p* = 0.072). Inhibitory taxa present on *L. nannotis* and *L. serrata* from the Kirrama uplands clustered more tightly than those on frogs at any other site (**Figure 5**; *post hoc* Benjamini and Hochberg adjusted *p*-values: Kirrama upland – Kirrama lowland, *p* = 0.024: Kirrama upland – Wooroonooran upland, *p* = 0.046 and Kirrama upland – Wooroonooran lowland, *p* = 0.046, all other pairwise comparisons, *p* > 0.05).

**FIGURE 6:**
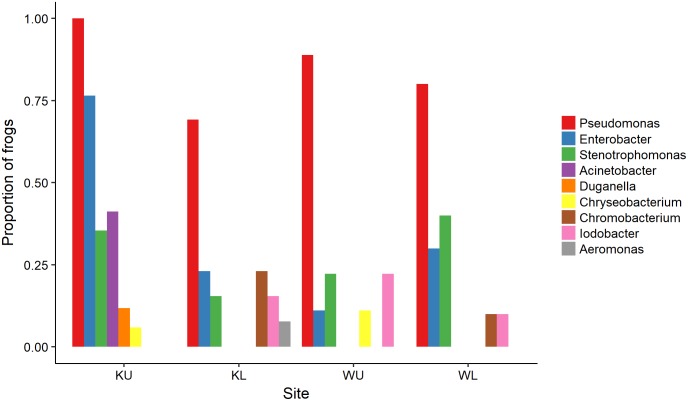
Proportion of frogs with isolates in each OTU by site. For ease of visualization, OTUs that occurred on fewer than two frogs at each site have been excluded. KU, Kirrama upland; KL, Kirrama lowland; WU, Wooroonooran upland; WL, Wooroonooran lowland.

The outcome of our GLM examining the number of *Bd*-inhibitory OTUs present on frogs as a Poisson-distributed response showed a significant interaction effect, indicating that site and species had effects that were not independent (ANOVA; χ^2^ = 44.938, df = 7, *p* < 0.0001). We therefore performed a *post hoc* analysis comparing 28 pairs of combinations of species and site (Tukey, 1949), using Benjamini and Hochberg adjusted *p*-values. This revealed that the differences among species by site were largely driven by *L. nannotis* at the Kirrama upland site. *L. nannotis* from the Kirrama upland site had significantly greater numbers of *Bd*-inhibitory bacterial OTUs than *L. nannotis* at all three other sites (for each site, *p* < 0.05) and significantly greater numbers of *Bd*-inhibitory bacterial OTUs than *L. serrata* at Kirrama lowland and upland sites (*p* < 0.05). There was also a suggestion of greater OTU numbers in *L. nannotis* from the Kirrama upland site compared with Wooroonooran upland *L. serrata* (*p* = 0.061). A full set of pairwise comparisons are provided as Supplementary Table 1. *Bd*-inhibitory members of the genera *Acinetobacter* and *Duganella* were unique to frogs at the Kirrama upland site (**Figure 6**).

**FIGURE 7:**
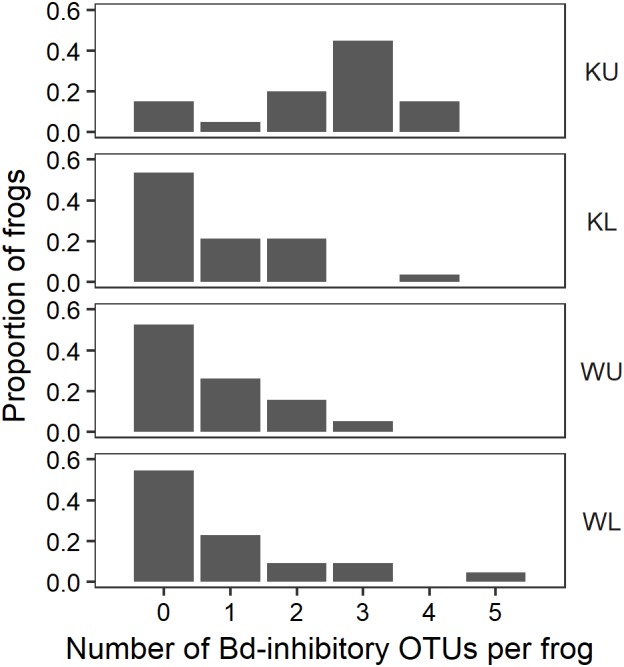
The proportion of frogs with *Bd*-inhibitory OTUs for each field site. KU, Kirrama upland; KL, Kirrama lowland; WU, Wooroonooran upland; WL, Wooroonooran lowland.

As well as having significantly more *Bd*-inhibitory bacterial OTUs on *L. nannotis*, the Kirrama upland site also had a significantly higher proportion of frogs with one or more *Bd*-inhibitory OTUs than other sites (**Figure 7**; Fisher’s Exact Test; *p* = 0.0497; *L. nannotis* and *L. serrata* only due to the absence of *L. rheocola* from the Kirrama upland site). When the Kirrama upland site was removed from the analyses, all other sites did not differ significantly from each other (Fisher’s Exact Test; *p* = 0.861). The proportion of frogs with *Bd*-inhibitory OTUs did not differ significantly between species (Fisher’s Exact Test; *p* = 0.797; *L. nannotis* and *L. serrata* only). This suggests that the site effect we detected was not a confounded effect of species.

## Discussion

We investigated the potential role that *Bd*-inhibitory cutaneous bacterial microbiota of Australian Wet Tropics frogs may have played in the reappearance of upland frog populations following population declines in the early 1990s and found that frogs from the Kirrama upland site differed from frogs at other sites in the presence and nature of their *Bd*-inhibitory bacteria. There was also a significant negative relationship between the upper limit of *Bd* infection intensity and the number of totally inhibitory bacterial genera on infected frogs. This suggests that greater inhibitory potential is linked to a reduction in the intensity of *Bd* infection, and that it therefore is possible that greater numbers of *Bd*-inhibitory bacterial genera may contribute to tolerance of *Bd* infection.

While our sampling effort was not ideal, it is often impossible for studies based on cultured isolates to achieve adequate sampling effort. For example, **Figure 3A** suggests that approximately 30 cultured totally inhibitory isolates per frog would be adequate. On average, our study found that 22% of the cultured isolates that we tested were totally inhibitory to *Bd*. Therefore, this would have required us to sample and test about 130 isolates from each frog/agar plate (more than 11,500 isolates from the 89 frogs in this study) in order to find 22% inhibitory. In almost all cases, there were considerably less colonies present on the agar plates than this and in many cases we sampled the majority of colonies that grew on the plate. In every case, we sampled all colony morphotypes present on the agar plates in an attempt to capture the diversity present. From our sequencing results, we often obtained identical sequences from individual frogs indicating that we probably over-sampled our agar plates, rather than under-sampled them. For these reasons, it is highly likely that we have captured the majority of cultured inhibitory OTUs present. The use of multiple culture media types may have resulted in the discovery of more OTUs, but it is likely that only a small fraction of all bacteria present on the frogs could ever be captured by culturing.

Many recent studies have used next generation sequencing techniques to investigate the diversity of amphibian cutaneous bacteria (reviewed by Jimenez and Sommer, 2017), but there is generally little evidence for a negative relationship between uncultured bacterial species richness and *Bd* infection load. However, uncultured proteobacteria phylotype richness was negatively correlated with *Bd* load in *E. coqui* frogs in Puerto Rico (Longo et al., 2015) and the relative abundance of certain taxa was negatively correlated with *Bd* infection load in *Rana sierrae* in California (Jani and Briggs, 2014). There are two possible reasons for negative relationships between *Bd* infection intensity and *Bd*-inhibitory OTU numbers. Frogs with naturally lower numbers of inhibitory bacterial taxa could be more susceptible to *Bd* or conversely, more intense *Bd* infection may cause a reduction in the numbers of inhibitory bacterial taxa.

*Bd* exposure can change the microbiome’s composition (Jani and Briggs, 2014; Walke et al., 2015b; Bataille et al., 2016; Jani et al., 2017). Studies have demonstrated that some OTUs can respond positively or negatively to *Bd* infection (Jani and Briggs, 2014; Familiar López et al., 2017; Longo and Zamudio, 2017) and that higher microbiome richness is associated with host persistence against *Bd* (Jani et al., 2017). However, increasing *Bd* load has not been demonstrated to lead to decreases in overall bacterial diversity or in the diversity of *Bd*-inhibitory bacteria on hosts. Laboratory experiments *in vivo* are therefore needed to ascertain whether a progressing *Bd* infection can cause a reduction in the richness of symbiotic bacterial communities, especially those inhibitory to *Bd*.

*In vitro* challenge assays have shown that combinations of multiple cultured bacterial strains are more effective at inhibiting *Bd* than are individual strains alone through additive and synergistic effects (Loudon et al., 2014; Piovia-Scott et al., 2017). This provides strong supporting evidence to our observations that increased numbers of inhibitory genera are associated with reduced susceptibility to *Bd.* Similar additive and synergistic effects have led to stronger fungal inhibition in hydra (Fraune et al., 2015), and soil Pseudomonads (Jousset et al., 2014). Three principal *Bd*-inhibitory genera (*Pseudomonas*, *Enterobacter*, and *Stenotrophomonas*) were present at all of our sites. The diverse secondary metabolites that can be produced by these principal genera might perform complementary roles. For example, one compound may act on the zoospore cell membrane while another interferes with transcription of DNA and therefore cell replication (Ghannoum and Rice, 1999; Kathiravan et al., 2012). It therefore seems possible that frogs in our study with greater numbers of *Bd*-inhibitory genera had more synergistic interactions among bacterial community members, leading to the production of additional antimicrobial metabolites and a reduction in either or both the probability of infection by *Bd* and the upper limits of infection load if infected (**Figure 4**).

Ideally, laboratory inoculation-infection experiments are needed to determine whether artificially increasing the number of *Bd*-inhibitory bacterial taxa on frog skin alters susceptibility to, or intensity of *Bd* infections. Similar experiments have been conducted on both plants and locusts and have demonstrated reduced pathogen density when a greater number of bacterial symbionts was present (Dillon et al., 2005; Matos et al., 2005; Hol et al., 2015) indicating the protective effect of a more diverse microbiome.

Our results from the Kirrama upland site showed that frogs at this site differed from all other sites in a number of ways, and together, provide support for protection against *Bd* infection by increased number of *Bd*-inhibitory bacterial genera. Frogs at this site had a significantly lower prevalence of infection than those at the Wooroonooran upland site, a significantly higher proportion of frogs with one or more *Bd*-inhibitory OTUs than other sites (though this could in part have been influenced by species effects), a higher proportion of inhibitory isolates than their conspecifics at other sites, significantly more *Bd*-inhibitory bacterial OTUs per frog in *L. nannotis* (suggesting increased synergistic anti-*Bd* potential), and statistically significant clustering of individual frogs with similar *Bd*-inhibitory taxonomic signatures (suggesting local selective pressure on skin microbiota). Some of the patterns we observed at the Kirrama upland site were clearly driven by *L. nannotis*. However, given that *L. serrata* only suffered temporary upland declines, it seems likely that the acquisition of greater numbers of *Bd*-inhibitory genera by *L. nannotis* at the Kirrama upland site may have facilitated their recolonization. A higher proportion of Kirrama upland frogs had *Bd*-inhibitory bacteria from the genera *Pseudomonas* and *Enterobacter* than at any other site, and the genera *Acinetobacter* and *Duganella* were unique to frogs from this site. Together, these differences suggest that Kirrama upland frogs, under pressure from enzootic *Bd* infection, have actively or passively acquired a greater number of *Bd*-inhibitory genera than frogs elsewhere, and that this may facilitate a reduction in the prevalence of *Bd* at this site.

All three species of frogs in this study have recolonized the Wooroonooran upland site despite having a lower proportion of *Bd*-inhibitory isolates than frogs at the Kirrama upland site and a lower proportion of frogs with *Bd*-inhibitory bacteria. Given the substantial genetic separation between frogs in the two regions (Schneider et al., 1998; Cunningham, 2001; Hoskin et al., 2005; Richards et al., 2010; Bell et al., 2012), it is likely that acquisition of resistance to *Bd* infection has evolved independently at the two upland sites. Therefore, factors other than symbiotic bacteria have probably played a part in aiding recolonization of the Wooroonooran upland site. These could include host immune defense, behavior, and environmental factors (Rollins-Smith et al., 2011; Savage and Zamudio, 2011; Rowley and Alford, 2013; Jani et al., 2017; McKnight et al., 2017; Voyles et al., 2018).

Bacteria that can inhibit the growth of *Bd* have received much attention in recent years, as it is possible that they could be cultured and used as probiotics to support at-risk populations of frogs through disease outbreaks (Becker et al., 2012; Bletz et al., 2013; Küng et al., 2014). Therefore the need to culture bacterial isolates to assess inhibitory potential against *Bd* remains essential to facilitate probiotic development, because it is not yet possible to accurately predict the functional capability of a community from 16S amplicon sequence data alone (Bletz et al., 2013; Becker et al., 2015). Fortunately, many of the more common OTUs described in 16S amplicon studies also appear to be culturable (Walke et al., 2015a) giving confidence that the functional capacity of the dominant community members was likely to be captured in our study. Future use of shotgun metagenomics, metatranscriptomics and metabolomics will enable insight into the functional capacity of bacterial communities without the need for culture (Rebollar et al., 2016) and help to elucidate the mechanisms by which individuals acquire and maintain communities of cutaneous bacteria that are inhibitory to *Bd*.

To our knowledge, we provide the first evidence that a greater number of *Bd*-inhibitory genera is correlated with lower *Bd* infection loads in wild frogs. This highlights the necessity for the creation of appropriate multiple-isolate combinations to promote potential synergistic interactions when developing probiotic candidates for supplementation of at-risk amphibian populations.

## Author Contributions

SB and RA designed the study, conducted the data analysis, and produced the first draft of the manuscript. SB completed the fieldwork. SB and SG processed samples in the laboratory. All authors edited the manuscript.

## Conflict of Interest Statement

The authors declare that the research was conducted in the absence of any commercial or financial relationships that could be construed as a potential conflict of interest.

## Footnotes

1 www.r-project.org
