# Supplementary material for: Increased Numbers of Culturable Inhibitory Bacterial Taxa May Mitigate the Effects of Batrachochytrium dendrobatidis in Australian Wet Tropics Frogs
Source: Front Microbiol. 2018 Jul 18;9:1604. doi: 10.3389/fmicb.2018.01604 (PMC6058028; doi:10.3389/fmicb.2018.01604)
Supplement: Supplementary file 1 [file Table_1.docx]

Supplementary Material

Increased numbers of culturable inhibitory bacterial taxa may mitigate the effects of *Batrachochytrium dendrobatidis* in Australian Wet Tropics frogs

Sara C. Bell^1,3*^, Stephen Garland^2,4^, Ross A. Alford^1^

^1^College of Science and Engineering, James Cook University, Townsville, QLD, Australia

^2^[College of Public Health, Medical & Veterinary Sciences](https://www.jcu.edu.au/college-of-public-health-medical-and-veterinary-sciences), James Cook University, Townsville, QLD, Australia

^3^[Current address: Australian Institute of Marine Science, Townsville, QLD, Australia](https://www.jcu.edu.au/college-of-public-health-medical-and-veterinary-sciences)

^4^[Currently](https://www.jcu.edu.au/college-of-public-health-medical-and-veterinary-sciences) not affiliated to an academic institution

*** Correspondence:** Corresponding Author: [saracbell@gmail.com](mailto:saracbell@gmail.com)

**Supplementary Table 1.** Post-hoc pairwise comparisons resulting from a GLM examining the number of *Bd*-inhibitory OTUs present on frogs among combined site-species pairs. Significance codes are: ‘**’ = 0.01, ‘*’ = 0.05 and ‘.’ = 0.1. Adjusted p values are reported using the Benjamini and Hochberg method (Benjamini and Hochberg, 1995). Species are *Litoria serrata* (LS) and *L. nannotis* (LN). Sites are Kirrama upland (KU), Kirrama lowland (KL), Wooroonooran upland (WU), and Wooroonooran lowland (WL).

| Site-Species pairwise comparisons | Estimate | Std.Error | z value | Pr(>\|z\|) | Significance |
| --- | --- | --- | --- | --- | --- |
| KL LS - KL LN | -0.77319 | 0.49355 | -1.567 | 0.21879 |  |
| KU LN - KL LN | 0.93156 | 0.32745 | 2.845 | 0.02488 | * |
| KU LS - KL LN | 0.14310 | 0.37893 | 0.378 | 0.73183 |  |
| WL LN - KL LN | -1.76644 | 0.75955 | -2.326 | 0.06142 | . |
| WL LS - KL LN | 0.54857 | 0.43363 | 1.265 | 0.32021 |  |
| WU LN - KL LN | -0.82198 | 0.57177 | -1.438 | 0.26346 |  |
| WU LS - KL LN | -0.26236 | 0.57177 | -0.459 | 0.69605 |  |
| KU LN - KL LS | 1.70475 | 0.44381 | 3.841 | 0.00296 | ** |
| KU LS - KL LS | 0.91629 | 0.48305 | 1.897 | 0.13496 |  |
| WL LN - KL LS | -0.99325 | 0.81650 | -1.216 | 0.32981 |  |
| WL LS - KL LS | 1.32176 | 0.52705 | 2.508 | 0.04251 | * |
| WU LN - KL LS | -0.04879 | 0.64550 | -0.076 | 0.93975 |  |
| WU LS - KL LS | 0.51083 | 0.64550 | 0.791 | 0.50018 |  |
| KU LS - KU LN | -0.78846 | 0.31140 | -2.532 | 0.04251 | * |
| WL LN - KU LN | -2.69800 | 0.72822 | -3.705 | 0.00296 | ** |
| WL LS - KU LN | -0.38299 | 0.37605 | -1.018 | 0.41128 |  |
| WU LN - KU LN | -1.75354 | 0.52944 | -3.312 | 0.00864 | ** |
| WU LS - KU LN | -1.19392 | 0.52944 | -2.255 | 0.06142 | . |
| WL LN - KU LS | -1.90954 | 0.75277 | -2.537 | 0.04251 | * |
| WL LS - KU LS | 0.40547 | 0.42164 | 0.962 | 0.42793 |  |
| WU LN - KU LS | -0.96508 | 0.56273 | -1.715 | 0.17269 |  |
| WU LS - KU LS | -0.40547 | 0.56273 | -0.721 | 0.52774 |  |
| WL LS - WL LN | 2.31501 | 0.78174 | 2.961 | 0.02144 | * |
| WU LN - WL LN | 0.94446 | 0.86603 | 1.091 | 0.38565 |  |
| WU LS - WL LN | 1.50408 | 0.86603 | 1.737 | 0.17269 |  |
| WU LN - WL LS | -1.37055 | 0.60093 | -2.281 | 0.06142 | . |
| WU LS - WL LS | -0.81093 | 0.60093 | -1.349 | 0.29184 |  |
| WU LS - WU LN | 0.55962 | 0.70711 | 0.791 | 0.50018 |  |

**References**

Benjamini, Y., and Hochberg, Y. (1995). Controlling the false discovery rate - A practical and powerful approach to multiple testing. *Journal of the Royal Statistical Society Series B-Methodological* 57, 289-300.

.
